# The Role of Radiomics in Lung Cancer: From Screening to Treatment and Follow-Up

**DOI:** 10.3389/fonc.2021.603595

**Published:** 2021-05-05

**Authors:** Radouane El Ayachy, Nicolas Giraud, Paul Giraud, Catherine Durdux, Philippe Giraud, Anita Burgun, Jean Emmanuel Bibault

**Affiliations:** ^1^ Radiation Oncology Department, Georges Pompidou European Hospital, Assistance Publique-Hôpitaux de Paris, Université de Paris, Paris, France; ^2^ Cancer Research and Personalized Medicine-Integrated Cancer Research Center (SIRIC), Georges Pompidou European Hospital, Assistance Publique-Hôpitaux de Paris, Université de Paris, Paris, France; ^3^ INSERM UMR 1138 Team 22: Information Sciences to support Personalized Medicine, Cordeliers Research Centre, Paris Descartes University, Paris, France; ^4^ Radiation Oncology Department, Haut-Lévêque Hospital, CHU de Bordeaux, Pessac, France

**Keywords:** radiomics, lung cancer, machine learning, oncology, lung cancer screening, treatment outcome and efficiency

## Abstract

**Purpose:**

Lung cancer represents the first cause of cancer-related death in the world. Radiomics studies arise rapidly in this late decade. The aim of this review is to identify important recent publications to be synthesized into a comprehensive review of the current status of radiomics in lung cancer at each step of the patients’ care.

**Methods:**

A literature review was conducted using PubMed/Medline for search of relevant peer-reviewed publications from January 2012 to June 2020

**Results:**

We identified several studies at each point of patient’s care: detection and classification of lung nodules (n=16), determination of histology and genomic (n=10) and finally treatment outcomes predictions (=23). We reported the methodology of those studies and their results and discuss the limitations and the progress to be made for clinical routine applications.

**Conclusion:**

Promising perspectives arise from machine learning applications and radiomics based models in lung cancers, yet further data are necessary for their implementation in daily care. Multicentric collaboration and attention to quality and reproductivity of radiomics studies should be further consider.

## Introduction

Death from lung cancer is estimated to be 1.7 millions each year worldwide, essentially due to late diagnoses (1), making it the first cause of cancer-related death in the world (2) despite recent discoveries in the field of tumor biology and new treatment strategies. The emergence of new targeted treatment focusing on specific biomolecular alterations such as EGFR (3) and ALK mutations has led to a new paradigm of cancer care, so-called “personalized” medicine, conversely to the historic “one-size-fits-all” medicine. In that regard, radiomics could also play a role in patient-specific treatment adaptations.

Common imaging interpretation, for instance with positron emission tomography (PET), Magnetic resonance imaging (MRI) or computed tomography (CT), relies on the visual analysis in terms of size, shape, signal intensity or contrast enhancement of various structures within the image.

« Radiomics », with reference to genomics, has been introduced in 2012 by Lambin et al. (4). Its aim is to extract a large number of quantitative variables from medical imaging, followed by a selection of the most informative ones in order to derive a scientific hypothesis.

Radiomics is based on the innovative approach that computerized algorithms are able to process imaging exams into more complex quantitative data. They can be applied to different imaging modalities (ultrasound, CT, PET, conventional radiology) by analyzing in a selected region of interest (ROI) the distribution of signal intensities.

Different ROI segmentation methods can be used. Manual delineation is close to daily practice, but requires a considerable amount of human time, limiting the creation of large databases, and is subject to high inter- and intra-observer variability (5–7). Automatic segmentation is thus largely preferable for reproducibility purposes, but is only applicable when there is a strong signal difference between the lesion and the adjacent tissues. This is why semi-automatic approaches are most often necessary: a software program defines a delineation which is then adjusted by the observer (8).

**Figure 1:**
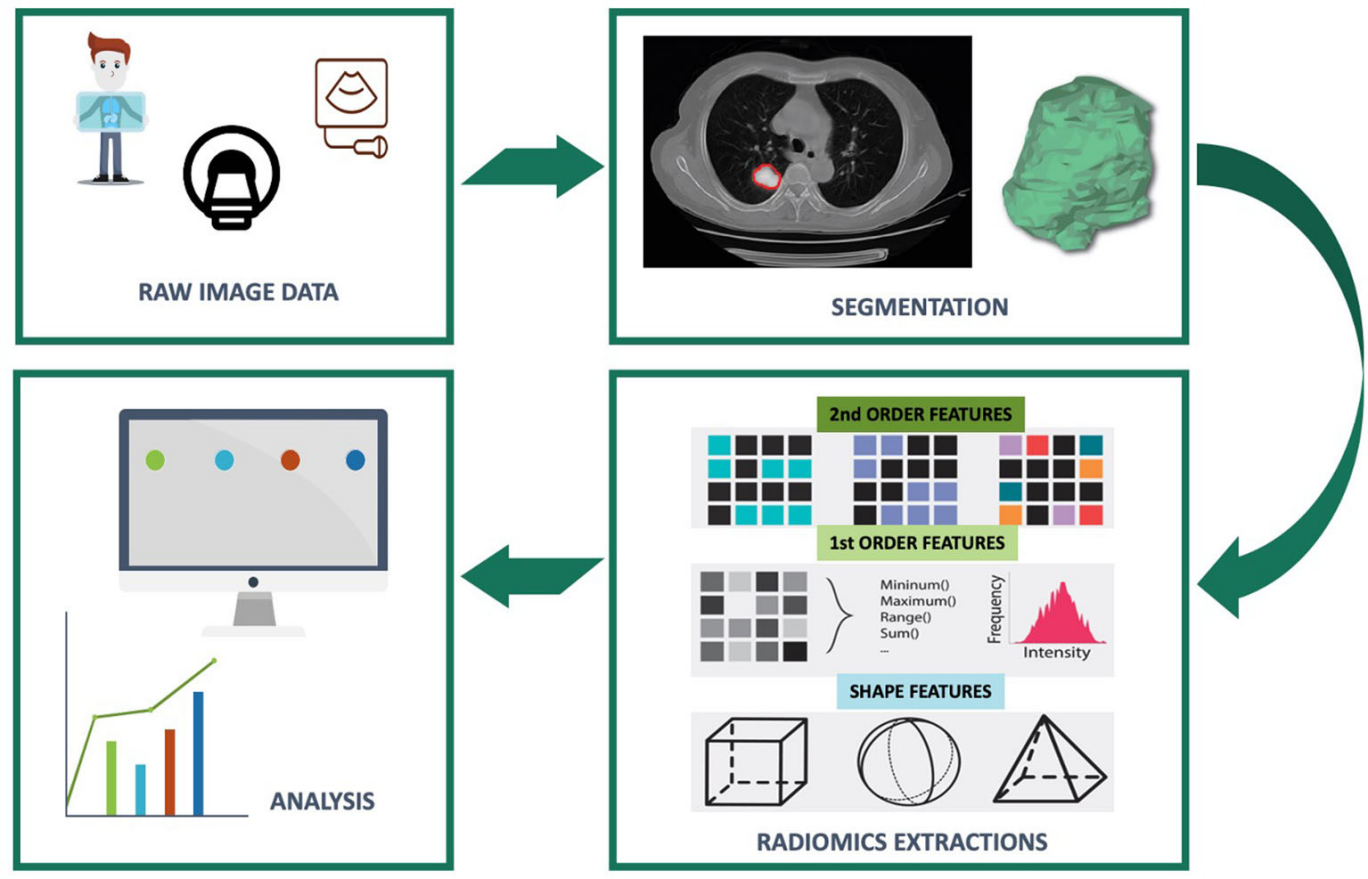
Flowchart of radiomics feature based analysis.

The extracted variables are divided into three categories (**Figure 1**): shape variables, first-order variables and second-order variables. The shape variables describe, independently of grey levels, the shape, surface area and dimensions of the ROI (example: surface area in square millimeters, sphericity,…). The first-order variables study the distribution of voxel gray level intensity values without consideration of spatial relationships. As for the second-order variables, they describe the spatial relationships between the voxels generally from matrices (example: grayscale co-occurrence matrix, size of homogeneous grayscale areas, neighborhood grayscale difference, length of grayscale ranges, grayscale dependence).

Like other high-throughput techniques, labeled “-omics” (9), radiomics aims to develop new imaging biomarkers to better understand the microbiology of cancer (10). The use of radiomics could provide additional data about the biological constitution of a tissue, predict treatment response or even offer new prognostic markers.

Radiomics thus offer several advantages due to their non-invasive character, the possibility to account for intra-tumor heterogeneity (11) by a complete analysis of the tumor, and inter-lesional heterogeneity (12) by sampling all the tumors within the same patient as well as the tumor microenvironment. They also allow monitoring temporal heterogeneity (13).

The last few decades have been paved by the advent of clinical, biological, radiological and genomic diagnostic advances offering access to a multitude of new data available for each patient as well as by the development of new therapeutics that are more targeted and personalized to each patient. Given the large amount of information generated, the major challenge in enabling personalized treatment in oncology lies in the ability to exploit this wealth of information to accurately predict the behavior and response of a tumor. Machine learning seems to be able to process and manage this huge amount of information.

In machine learning, a classification model is trained from a data set in order to “learn” (training set) the distribution of the different classes in a multidimensional variable space. In machine learning, there are several methods, each with their advantages and disadvantages (14). They are grouped into two types of classification: supervised and unsupervised.

In the supervised classification methods, individuals are labelled (e.g., benign vs. malignant) and the algorithm tries to predict this explicit variable, called the output variable, from a large number of input variables (radiomics, genomics, clinical,…).

Unsupervised methods do not use predefined output variables. The goal is to find a model that groups the most similar data together and separates the most different data, known as clustering. For example, K-means clustering generates K clusters by comparing the degree of similarity of observations, so that two individuals that are similar will have a reduced distance of dissimilarity.

**Figure 2:**
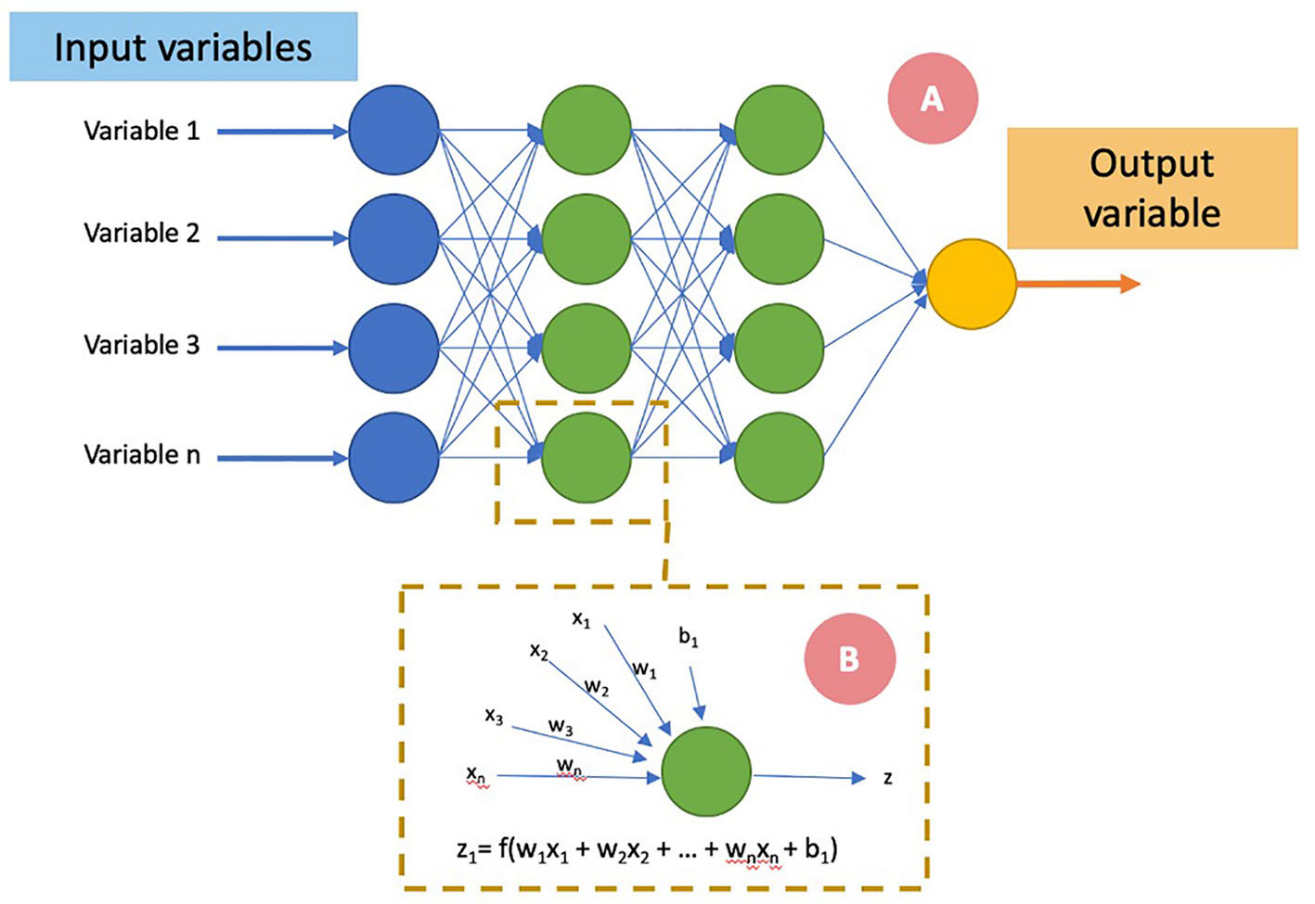
Schematic representation of an artificial neural network. The input variables **(A)** are presented at the first neural layer (blue). The information is then passed to a succession of layers (“hidden layers,” in green) and finally an output neural layer predicting the variable to be estimated. Each layer (i) consists of Ni neurons, taking their inputs from the Ni-1 neurons of the previous layer. A neuron **(B)** adds each of its inputs (xn) and multiplies them by a weight (wn). An activation function (f) allows according to a threshold the activation of the neuron and the transmission of information (z) to the next layer. An optimizer adjusts the weights and biases (b) of each neuron in order to make the neural network converge toward its state allowing it to make the best prediction.

One of the most used ML subset is Artificial Neural Network (ANN) (**Figure 2**). It is considered as a supervised classification model. Its variant, Deep Learning (DL), is associated with the feature extraction, directly from raw imaging data, through a series of nonlinear processing units comprising multiple layers, which tries to establish a relationship between stimuli and associated neural responses present in the brain.

The expansion of medical imaging data (14) in lung cancer offers an opportunity to explore the value of radiomics for every step of the patient’s care: screening, diagnosis, staging, treatment planning, and response evaluation. The objective of this article is to benchmark radiomics applications in lung cancer at each of these steps.

## Materials and Methods

The authors conducted a literature review using PubMed/Medline in order to identify important recent publications to be synthesized into a comprehensive review of the current status of radiomics in lung cancer at each step of the patients’ care. A comprehensive list of MeSH terms and keywords was included in the search: “lung cancer,” “radiomics,” “signature,” “machine learning,” as well as other associated technical ML keywords. Selected articles were published between January 2012 and June 2020, and based on relevance to the subject. The search strategy also included screening of reference lists of relevant publications. The search query returned 133 articles that were screened. We removed review articles and selected 49 studies in the final analysis.

## Results

### Characterization of Lung Abnormalities

One of the first application of radiomics in lung cancer was tumor detection. Lung abnormality discoveries are frequent; thus, the challenge is to be able to distinguish benign lesions from malignant ones. Qualitative features such as measurements of diameter or volume of pulmonary nodules provide important information to differentiate benign from malign nodules. Notwithstanding the encouraging results of low-dose computed tomography (CT) versus (vs.) chest X-ray in lung cancer-specific mortality reduction (15), the application of low-dose CT in selected population screening remains contested (16) on account of its cost-efficiency, the high false positive rate (FPR) and the optimal schedule (1). In that setting, overdiagnosis remains a challenging issue (17). In addition, due to the lack of validated software, the volumetric assessment of the lesion is not the current standard of practice (18). 18F-fluorodeoxyglucose positron emission tomography (18F-FDG PET)/CT is a performant tool to help clinicians in the characterization of lung nodules (19) but still holds a low detection rate of small lesions (20) and delivers high radiation doses.

Recent promising strategies based on radiomics or circulating biomarkers (21) could be interesting and less invasive (22). Computer aided diagnosis (CAD) systems can help to improve radiologists’ performances (23) on tumor detection and could be even further improved by radiomics.

Radiomics features could be used in traditional statistic model as linear classifier with high accuracy in predicting lung nodule malignancy (24).

**Figure 3:**
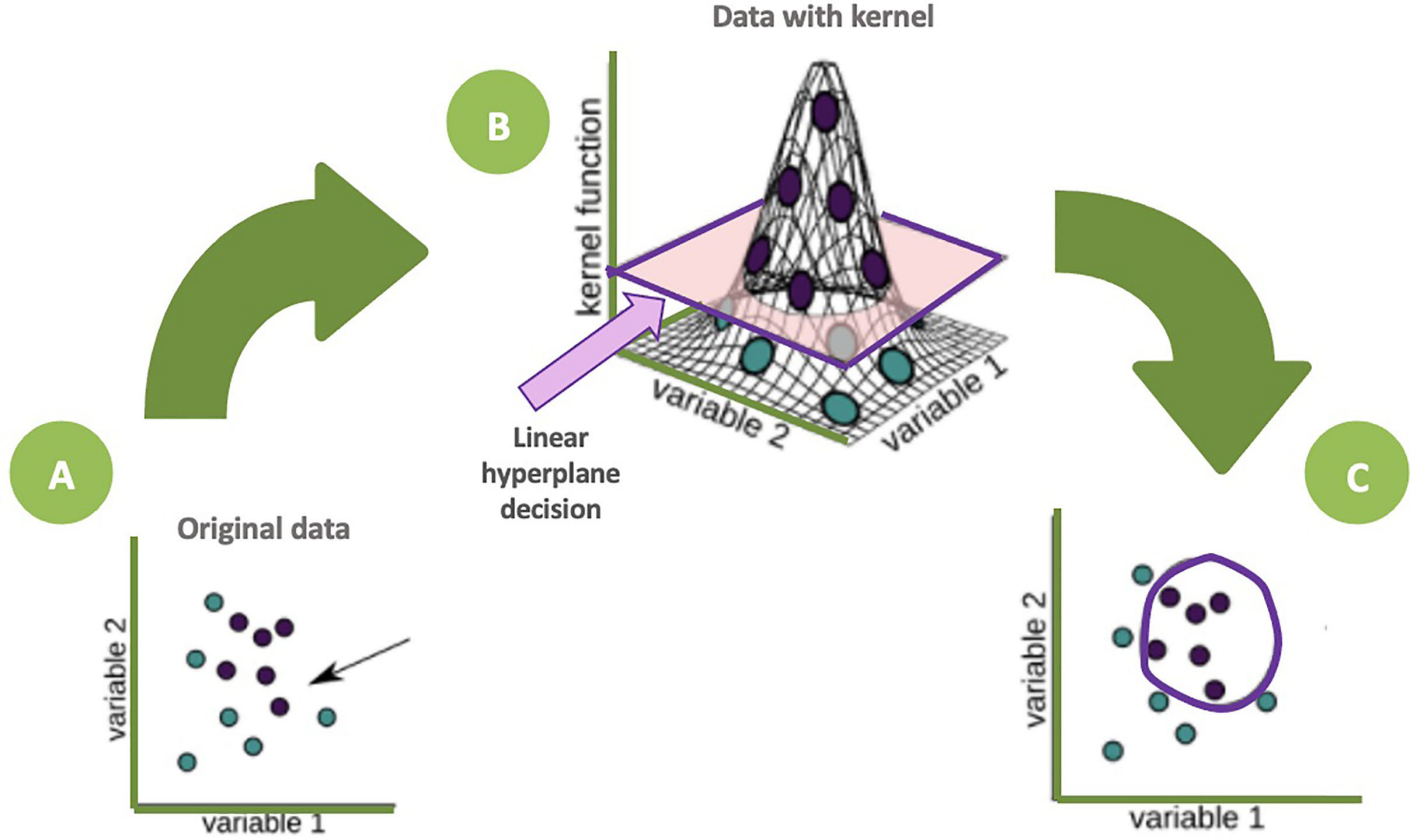
Schematic representation of a SVM algorithm. The dots represent individuals according to two variables **(A)**, no linear classification function seems obvious. The kernel function allows a representation of the individuals in a 3rd dimension allowing the highlighting of a hyperplane which classifies the individuals in two groups **(B)**. The individuals are then projected into the initial dimensional space **(C)** with a non-linear separator (purple circle).

Integrating radiomics, the optimal ML model to apply remains unknown. Random forest classifiers showed good performance in anticipating nodules that would become cancerous one and two years later, with accuracies of 80% (25), better than a Support Vector Machine (SVM) classifier or the recently developed McWilliams (26) and Lung-RADS (27) risk scores. Schematically, SVM models, through a kernel function, depict individuals in a 3^rd^ dimensional space in order to find a hyperplane that classifies individuals into two groups (**Figure 3**).

Different supervised ML models can also be used together. After a feature selection by a Random Forest classifier (RFC), Wang et al. (28) found 15 radiomics features able to single out benign from malignant nodules with an accuracy of 86% through a SVM algorithm.

Some studies tried to benchmark the added value of clinical features to these radiomics features. As a matter of fact, they can improve the performance of ML methods to distinguish focal pneumonia from adenocarcinoma (29) or non-small-cell lung cancer (30). Clinical features addition could also produce no improvement of the model performance (24), highlighting the importance of the radiomics features.

Interestingly, some studies (31) indicate a trend toward increased performance when the surrounding parenchyma is included, revealing the importance of microenvironment.

Most studies use radiomics approaches needing 2D or 3D quantitative images features. Another category of computational strategy is Deep learning and particularly convolutional neural networks (CNN). CNN could perform prediction without needing nodule segmentation, taking directly as an input the raw imaging data. Deep learning showed good performance for differentiating lung nodule from other thoracic structures (vessels, bone, …) (32–37). Particularly in a study, Causey et al. (38) processed 1065 nodules with different malignancy scores. The model was developed with a deep CNN architecture, capable of performing classification or producing a feature vector that could then be used as input to a secondary classifier such as a RFC. The CNN classification highly performed (AUC 0.97) and was improved (AUC 0.99) when combined to handcrafted radiomics features (38) through a RFC. The main studies relating to lung nodule classification are summarized in **Table 1**.

**Table 1:** Mains studies regarding lung nodule prediction of malignancy.

Reference	Number of cases	Imaging modality	Algorithm	Segmentation	Feature types	No of features	Validation	Results
Hawkins et al. (25)	598	CT	RFC	Semi-automatically segmented	Shape ++, 1^st^ order	23	Cross-validation	AUC 0.83 at 1 year
Balagurunathan et al. (24)	479 (244 for Training)	CT	Linear classifier	Semi-automatically segmented	Shape, 1^st^ order, 2^nd^ order	4	Split sample	AUC 0.83
Wang et al. (28)	593 (400 for Training)	CT	SVM	Semi-automatically segmented	Shape, 1^st^ order, 2^nd^ order	15	Split sample	Accuracy 86%
Chen et al. (39)	72	CT	SVM	Manually segmented	Shape, 1^st^ order, 2^nd^ order	4	Cross-validation	Accuracy 84%
Dilger et al. (31)	50	CT	ANN	Manually segmented + surrounding lung parenchyma	Shape, 1^st^ order, 2^nd^ order	5	Cross-validation	AUC 0.938
Causey et al. (38)	1065	CT	CNN + RFC	Semi-Automatic + manually segmented radiomics	Deep features	NE	Split sample	AUC 0.99

ANN, artificial neural network; AUC, area under the curve; CNN, convolutional neural network; CT, computed tomography; NE, not evaluable; PSO, particle swarm optimization; RFC, random forest classifier; SVM, support vector machine.

Major hope is that characterization of lung abnormalities could potentially allow for an early diagnosis of lung cancer, even for very small nodules, aiming to considerably improve patients’ prognosis.

### Histology and Radio-Genomics

When a suspicious lung abnormality is detected on imaging, obtaining histological evidence of cancer is necessary. It often requires an invasive procedure, sometimes leading to technical difficulties or complications. Thus, some patients, due to their state of health, are unable to undergo a biopsy.

Radiomics provide a promising alternative in this regard. From CT exam, radiomics features could be extracted to characterize tumor histology. From two independent cohorts, a Naïve Baye’s classifier achieved a high AUC (0.72; *p*-value = 2.3 × 10^−7^) with only five features (40).

Using ANN, similar performances for the prediction of histopathology were also obtained. Raniery Ferreira et al. (41) constructed different machine learning models for histopathological pattern recognition. From a dataset of 68 malignant lung tumors with confirmed histology, they extracted radiomics features by a semi-automatically segmentation. The radial basis function-based (RBF) ANN obtained an AUC of 0.71 on histopathological pattern recognition with radiomics features. In this study, adding clinical to radiomics features provided different behaviors on the models’ performances on the testing and validation sets, and did not improve the results.

This last decade, targeted treatments played a leading role in lung cancer management (42). For most of those treatments, the identification of a specific mutation requires an invasive biopsy of the tumor, not always performable thus potentially depriving these patients of highly beneficial treatment. A more recent alternative could be liquid biopsy, consisting of the search of mutations on circulating tumor cells or DNA by a blood sample. Liquid biopsy has recently demonstrated its clinical usefulness in advanced NSCLC but keeps very poor sensitivity in early stage lung tumors (43, 44). The most common gene mutations seen in non-small-cell lung carcinoma (NSCLC) are V-Ki-ras2, Kirsten rat sarcoma viral oncogene homolog (KRAS), epidermal growth factor receptor (EGFR), v-raf murine sarcoma viral oncogene homolog B1 (BRAF), and anaplastic lymphoma kinase (ALK); of these, KRAS and EGFR mutations are the most commonly detected.

Regarding the specific mutation identification, the association of clinical features with radiomics ones seems to provide added value. Zhang et al. (45) conducted a multivariate analysis using seven handcrafted radiomics and three clinical features of 180 cases. They predicted EGFR mutation with an AUC of 0.87. Another study (46) explored a multicentric CT dataset of 381 patients who underwent surgical resection. The 20 remaining radiomics features using a RFC outperformed good prediction in discriminating between EGFR+ and EGFR- tumors (AUC 0.69). A clinical model of EGFR status (AUC 0.70) was combined to significantly improve prediction accuracy (AUC 0.75). The highest performing signature was capable of distinguishing between EGFR+ and KRAS+ cases (AUC 0.80) and, when combined with a clinical model (AUC 0.81), substantially improved its performance (AUC 0.86). One study by Zhao et al. (47) aimed at predicting EGFR mutation status and subtypes, in particular the two most common ones (exon 19 deletion and exon 21 L858R mutations). A radiomics score (R-score) based on 11 radiomics features was calculated for each lesion. Using a radiomics-based model and a combined radiomics and clinical model, the respective AUC values in the validation cohort were 0.73 and 0.76.

Deep learning methods have also been explored in prediction of genomic alterations. Using a CNN-based approach, Wang et al. (48), by training a network on 14926 CT images from 603 patients, achieved encouraging predictive performance on a validation cohort of 241 patients (AUC 0.81). For applying the deep learning model, a cubic region of interest (ROI) containing the entire tumor was manually selected. The first 20 convolutional layers were trained using transfer learning by 1.28 million natural images from the ImageNet dataset avoiding as much as possible an overfitting and the last four convolutional layers were trained using CT images from lung adenocarcinoma tumors in the independent test cohort. Authors used a method to visualize tumor region that was most related to EGFR mutation status.

While these studies focused on CT-based radiomics, another imaging modality commonly used in oncology is PET-CT. In PET-based radiomics, radiomics features could detect EGFR mutation status with good performance. Zhang et al. (49) developed a radiomics signature made of 10 features (PET and CT radiomics features) trained on 175 patients. The model showed a significant ability to discriminate between EGFR mutation and EGFR wild type in the validation set (AUC 0.85), which was improved when combined with clinical variables (AUC 0.87).

Yamamoto et al. (50) aimed instead at predicting the ALK status using visual qualitative CT features combined with clinical parameters. Their predictive model had a good performance in both the training and the validation set. Another study including clinical and radiomics variables extracted from PET and CT (51) from 539 patients with confirmed lung adenocarcinomas investigated the potential of differentiating the ALK/ROS1/RET fusion-positive and fusion-negative adenocarcinomas, building a model that resulted in 73% sensitivity and 70% specificity with seven features.

The main studies dealing with histologic and radio-genomics prediction are summarized in **Table 2**.

**Table 2:** Mains studies regarding histology and radio-genomic characterization.

Reference	Application	Number of cases	Imaging modality	Algorithm	Segmentation	Feature types	No of features	Validation	Results
***Histology subtypes***									
Wu et al. (40)	Prediction of histology subtype	350 (198 for Training)	CT	Naïve Baye’s classifier	Manually segmented	Shape, 1^st^ order, 2^nd^ order	5	Independent	AUC 0,72
Raniery Ferreira et al. (41)	Prediction of histology subtype	68 (52 for Training)	CT	RBF-based ANN	Semi-Automatically segmented	Shape, 1^st^ order, 2^nd^ order	100	Sample split	AUC 0,71
***Genomic alterations***									
Zhang et al. (45)	Prediction of EGFR mutation	180 (140 for Training)	CT	multivariate analysis	Manually segmented	Clinical, Shape, 1^st^ order, 2^nd^ order	7	Sample split	AUC 0,87
Velazquez et al. (46)	Prediction of EGFR and KRAS mutation	381 (190 for Training)	CT	RFC	Manually segmented	Clinical, Shape, 1^st^ order, 2^nd^ order	25	Independent	AUC 0,86
Zhao et al. (47)	Prediction of EGFR subtype	637 (322 for Training)	CT	multivariate analysis	Manually segmented	Clinical, Shape, 1^st^ order, 2^nd^ order	11	Sample split	AUC 0,76
Wang et al. (48)	Prediction of EGFR mutation	843 (603 for Training)	CT	CNN	Manual segmentation	Deep features	NE	Independent	AUC 0,81
Zhang et al. (49)	Prediction of EGFR mutation	248 (175 for Training)	PET, CT	Logistic regression	Semi-Automatically segmented	Clinical, Shape, 1^st^ order, 2^nd^ order	13	Sample split	AUC 0,87
Yoon et al. (51)	Prediction of ALK status	539	PET, CT	Logistic regression	Semi-Automatically segmented	Clinical, Shape, 1^st^ order, 2^nd^ order	7	Cross validation	sensitivity and specificity, 0.73 and 0.70, respectively

ALK, anaplastic lymphoma kinase; ANN, artificial neural network; AUC, area under the curve; CNN, convolutional neural network; CT, computed tomography; EGFR, epidermal growth factor receptor, KRAS, Kirsten rat sarcoma viral oncogene homolog, NE, not evaluable; PET, positron emission tomography; RBF, radial basis function; SVM, support vector machine.

### Treatment Outcome

Radiomics could play a role in predicting the prognosis and the treatment response, in order to adapt treatment strategies individually with view of personalized medicine. The main studies relating to this subject are summarized in **Table 3**.

**Table 3:** Main studies evaluating radiomics in prediction of treatment outcomes in lung cancer.

Reference	Application	Number of cases	Imaging modality	Feature selection method	Model algorithm	Segmentation	Feature type	No. of features	Validation	Results
***Radiotherapy***										
Dissaux et al. (52)	Local control after SBRT	87 (64 for Training)	CT – PET/CT	Univariate analysis	Multivariate regression	Semi-automatically + manually	1^st^ order, 2^nd^ order	2 (PET)	Independent set	Accuracy 0.91
Huynh et al. (53)	Outcomes after SBRT	113	CT	PCA	Concordance index	Manually	Clinical	15	Cross-validation	C-index of 0.33 for OS(q = 0.0016)
Zhang et al. (54)	Outcomes after SBRT	112	CT	PCA	RFC	Manually	1^st^ order, 2^nd^ order	NA	NA	OS: AUC 0,77
Yu et al. (55)	Outcome of stage I NSCLC	442 (147 for Training)	CT	Random Survival Forest	Multivariate regression	Manually	1^st^ order, 2^nd^ order	2	Independent set	OS: log-rank p=0.0173; HR 1.02, p= 0.0438
Hawkins et al. (56)	Outcome of NSCLC	81	CT	Relief-f	Decision tree	Manually	Shape, 1^st^ order, 2^nd^ order	5	Cross-validation	Accuracy 0.78
Aerts et al. (57)	OS of NSCLC and H&N cancer	1019 (474 for Training)	CT	Univariate analysis	Multivariate regression	Manually	Shape, 1^st^ order, 2^nd^ order	4	Independent set	C-index 0.65
Hosny et al. (58)	OS outcome of stage I and II NSCLC	1194 (786 for Training)	CT	NE	CNN	Manually	Deep features	NE	Independent set	AUC 0.71 and 0.70 for radiotherapy and surgery sets
Mattonen et al. (59)	Differentiate early recurrence from RILI post SBRT	45	CT at 3 months post SBRT	LOOCV	SVM	Semi-automatically	1^st^ order, 2^nd^ order	5	Cross-validation	AUC 0.85
Liang et al. (60)	Prediction of radiation pneumonitis	70	CT with dose distribution	Multivariate regression	Multivariate regression	Automatically	2^nd^ order	2	None	AUC 0,78
Coroller et al. (61)	Predict pathological response after chemoradiation	127	CT	PCA	Multivariate regression	Manually	Clinical, Shape, 1^st^ order, 2^nd^ order	10	Cross-validation	AUC 0.68
Lou et al. (62)	Local control after SBRT	944 (849 for Training)	CT	NE	CNN	Manually	Deep features, clinical (dose)	NE	Independent set	C-index 0.77
***Systemic treatment***										
Khorrami et al. (63)	Response to 1^st^ line chemotherapy	125 (53 for Training)	CT	LASSO	QDA	Manually	Shape, 2^nd^ order	7	Split sample	AUC 0.77
Kim et al. (64)	Response to 1^st^ line EGFR TKI	48	CT	Univariate analysis	Multivariate regression	Manually	Clinical, Shape, 1^st^ order, 2^nd^ order	5	None	C-index 0.77
Sun et al. (65)	Outcome anti-PD-1 and anti-PD-L1 treatment	272 (135 for Training)	CT	Elastic-net regularized regression	Elastic-net regularized regression	Semi-automatically	Location, technical, Shape, 1^st^ order, 2^nd^ order	8	Independent set	OS : HR 0.52; p=0.0022

AUC, area under the curve; CNN, convolutional neural network; CT, computed tomography; LASSO, least absolute shrinkage and selection operator; LOOCV, leave-one-out cross validation; NE, not evaluable; OS, overall survival; PCA, Principle Component Analysis; PET, positron emission tomography; QDA, Quadratic discriminant analysis; RFC, Random Forest Classifier; SVM, support vector machine.

#### Radiotherapy

In locally advanced lung cancer, radiotherapy, often associated with systemic therapies, is the standard option. A specific radiation option of lung cancer treatment is stereotactic body radiation therapy (SBRT), in inoperable patients presenting with a small local lesion (66). Radiosensitivity varies to a great extent across tumor types and also between patients bearing the same type of tumor. Biomarkers predicting the clinical outcome after radiotherapy are already available, but their levels of evidence are heterogeneous (67).

Radiomics features could be leveraged to predict different outcomes that conventional imaging metrics cannot predict in SBRT patients (68).

Several studies tried to predict different clinical endpoints such as local control and/or disease free survival and/or overall survival (52–58, 61) with good accuracy. Some others attempted to predict radiation induced toxicity (69), in particular to differentiate local failure from radiation induced lung injury (RILI) (59, 70).

Many of those studies outperformed different models concomitantly. Those studies revealed that a same feature selection technique and/or a same classifier model could considerably perform differently in distinct cohorts, suggesting a dependency not on the endpoint but on the study population.

The number of selected features is also notably heterogeneous between the studies from two (52) to fifteen radiomics features (53). After different feature selection methods, the texture features (i.e. second-order radiomics features) seemed to be the more correlated to clinical endpoints (53, 55, 71). Aiming to reduce the number of radiomics features, Diassaux et al. (52) found, in a multicentric study including 87 patients with an independent test set, a radiomics signature combining one PET feature and one CT feature predicting local control with an accuracy of 98%. They used ComBat harmonization method (72) on radiomics features to handle the differences of imaging acquisition. This method was initially used in gene expression microarray data to deal with the “batch effect,” i.e., the source of variations in measurements caused by handling of samples by different laboratories, tools and technicians. The advantage of this technique is that it allows a correction to be applied directly to the extracted radiomic variables as opposed to the images before extraction, making it easier to analyze retrospective and multicentric data.

In radiation oncology, total dose and space dose distribution are carefully evaluated for each patient during treatment planning. In that way, a study (62) queried the lung CT-derived feature space to identify radiation sensitivity parameters that can predict treatment failure and hence guide the individualization of radiotherapy dose. The authors input pre-therapy lung CT images into Deep Profiler, a multitask deep neural network that has radiomics incorporated into the training process. Then, they combined these data with clinical variables to derive *i*Gray, an individualized radiation dose that results in an estimation of failure probability below 5% at 24 months. Thus, it would seem that a reduction in the irradiation dose could have been proposed in 23.3% of patients.

Integration of reported dosimetric features from the dose distribution in the irradiated lung calculated in the planning CT, showed to be predictive of radiation pneumonitis (73). Liang et al. (60) used the “dosiomics” method, which attempts to extract the spatial features from dose distribution, for the occurrence of grade 2 or more RP prediction.

To assist the physician during treatment planning, visualization of high-risk tumor spot of treatment failure could be very convenient. In a study (74), the authors visualized which regions in the patient images predicted low survival probability. From such observations, the heat map visualization has the potential to identify regions at high risk for tumor progression or recurrence that could be utilized for the purpose of assisting patient-tailored treatment planning in the future.

During radiation therapy treatment and follow-up, patients are subject to several imaging procedures. Like blood circulating biomarker changes during treatment could be predictive of the effectiveness of some treatments (75), the question of radiomics features modification has been studies, called “delta radiomics.” It aims to analyze radiomics features’ evolution through time and treatments based of evaluations obtained from longitudinal scans. Some studies demonstrated that delta radiomics seem to be more robust than radiomics features with the potential of using delta features for early assessment of treatment response and developing tailored therapies (76). A study focusing on 107 patients with stage III NSCLC (77) tried to evaluate the impact of radiomics features changes due to radiation therapy and their values at the end of treatment on tumor response. All of the radiomics features changed significantly during radiation therapy. For local recurrence, pretreatment imaging features were not prognostic, while texture-strength measured at the end of treatment significantly stratified high- and low-risk patients.

Another study focused on Cone Beam CT (CBCT), commonly used in radiotherapy for patient’s precise setup, In this study, delta radiomics revealed to be predictive of overall survival in locally advanced lung cancer in a preliminary study with 23 patients (78).

In a study (79) including 268 patients with stage III NSCLC and using different CT at different timepoints of the treatment (pre-treatment, at 1, 3, and 6 months of follow-up), a deep learning networks was built to predict clinical outcomes of patients. Model performance was enhanced with each additional follow-up scan into the CNN model (2-year overall survival: AUC 0.74, p< 0.05).

In terms of toxicity prediction, Moran et al. (71) in a study with 14 patients who underwent SBRT tried to demonstrate the potential of CT-based radiomics on 3, 6 and 9 month post-SBRT CT to distinguish moderate/severe lung injury from none/mild lung injury. Texture features outperformed the first-order features in differentiating lung injury severity levels.

#### After Systemic Treatments

While early-stage lung cancer patients with large tumors (stage IB-IIA) who have undergone surgery are likely to receive adjuvant chemotherapy (68), inoperable patients or patients presenting locally advanced lung cancer often have co-morbidities that limit their tolerance to systemic treatment. Consequently, systemic treatments cannot be a generalizable recommendation for all patients. The advent of immunotherapy and targeted therapies over the last decade in the management of metastatic lung cancer has led to important clinical results with a very acceptable safety profile (80, 81). It is therefore more than necessary to be able to determine in advance which patients are at risk of not responding to therapy and thus allow either an intensification of the therapeutic strategy or of the therapeutic sequence or, conversely, avoid harmful therapies without benefit to the patient.

Radiomics showed good hope to be able to respond to this issue. In a study (63) including patients who were treated with front-line platinum-based chemotherapy, the combination of the top seven discriminating features outperformed an accuracy of 0.77 in prediction of tumor response. A significant correlation with both time to progression and overall survival for patients with NSCLC was also found.

Radiomics models could identify responders to EGFR tyrosine kinase inhibitor (TKIs) such as Gefitinib from the change in features between the pre-treatment and 3 weeks post-treatment CT. In a study conducted by Aerts et al. (82) including 47 patients, one delta-radiomics feature was significantly predictive (AUC 0.74) of Gefitinib response.

Pretreatment contrast-enhanced CT and first follow-up CT after initiation of EGFR TKIs were retrospectively analyzed in 48 NSCLC patients (64).

A recent promising treatment is immune checkpoint blocker (i.e. immunotherapy). The choice of patients who would benefit most from this treatment remains unclear and it is necessary to identify the good responders. Radiomics should have a role to play in this purpose (83).

Similarly, PET and PET/CT have been used for the prediction of treatment response. Radiomics signature was successfully validated to discriminate immune phenotype and predict survival and response to anti-PD-1 or PD-L1 immunotherapy (65, 84, 85).

Regarding the treatment sides effects, radiomics has been proven to be able to predict pneumonitis following immunotherapy (86), allowing closer surveillance for at-risk patients or even impacting the therapeutic choice.

## Discussion

This last decade, studies about radiomics drastically increased in different domains of oncology (87) with significant improvements. The new paradigm of precision medicine supports the research of new biomarkers and thus a lot of studies tried to explore radiomics in various applications with promising results which could have a huge impact on clinical routine. Machine and deep learning algorithms provide powerful modeling tools to explore the big amount of image data available, especially in oncology, to bring to light underlying complex biological mechanisms, and make personalized precision cancer diagnosis and treatment planning possible.

We could imagine a CAD, based on imaging, that directly establishes the nature of a lung lesion, its genomic alterations and provide guidance to physicians to choose the best therapeutic options that fit the most for each patient. Thus, the time between diagnosis and treatment initiation could be considerably reduced as well as the invasiveness of the procedures in patients who are, in most cases, very fragile. Patients could be offered therapeutics that are as effective as low in toxicity. For instance, SBRT, which is a first-choice treatment option for patients with stage I lung cancer who have surgical contraindications (88), could be proposed more broadly on the condition that the patients who can benefit from it could be accurately identified.

Then, in analogy to genomic signatures in breast cancer (89), therapeutic de-escalation may be possible when treatment would be identified as bringing no over gain.

Traditionally, the radiomics features being extracted are hand-crafted. Feature-based methods require a segmentation of the region of interest through a manual, semiautomated, or automatic methods. Then, hundreds or even thousands of radiomics features are extracted. Thus, feature selection and extraction are crucial steps that aim at obtaining the optimal feature representation that correlates most with the endpoint and correlates least between each other. Hand-crafted features suffer from the tedious designing process and may not faithfully capture the underlying imaging information. Semiautomatic segmentation could improve the stability of radiomics features (8) and fully automatic segmentation tools could be as accurate as manual segmentation by medical experts (90). With the development of deep learning based on multilayer neural networks, particularly CNN, the extraction of machine learnt features is becoming widely applicable. In deep learning, the processes of data representation and prediction are performed jointly (91). Pixel/voxel-based ML (PML) emerged in medical image analysis (92), which use pixel/voxel values in images directly instead of features calculated from segmented objects as input information; thus, feature calculation or segmentation is not required. Because the PML can avoid errors caused by inaccurate feature calculation and segmentation, the performance of the PML can potentially be higher than common classifiers. Moreover, the data representation removes the feature selection portion eliminating associated statistical bias in the process. The peritumoral space around the tumor may also provide valuable information over the visible tumor features for patient risk stratification due to cancer metastasis as demonstrated in a study carried by Dou et al. on 200 patients (93). A SVM classifier predicted distant failure with an accuracy of 0.83 thanks to analysis of the peritumoral space radiomics features from PET images of 48 NSCLC patients and 52 cervical cancer patients (94), arguing the fact that information around the tumor could provide better accuracy. PML are generally taking into account the peritumoral space. In a study evaluating a CNN based model (58), the visual mapping demonstrated that tissue within and beyond the tumor were both crucial for characterization and eventual prediction. CAD could be so able to highlight specific spot to overtreat.

It is clear that to this day, daily clinical radiomics applications remains very limited (95). At the present time, no clinical application of radiomics is available. Many factors could explain this situation (96).

First, the overall scientific quality and reporting of radiomics studies is insufficient. Scientific improvements need to be made to feature reproducibility, analysis of clinical utility, and open science categories. The Transparent Reporting of a multivariable prediction model for Individual Prognosis Or Diagnosis (TRIPOD) checklist (97) was adapted to radiomics studies by Park et al. (98) after finding very poor results of his analysis of multiple studies in term of radiomics quality scores and adherence to the TRIPOD checklist. It intends to improve the transparency of a prediction model study’s reporting regardless of the study methods. It is a checklist of 22 items considered important for good reporting of studies developing or validating multivariable prediction models. The items relate to the title and abstract, background and objectives, methods, results, discussion, and other information. The TRIPOD Statement covers studies that report solely development, both development and external validation, and solely external validation (with or without model updating) of a diagnostic or prognostic prediction model. Recently, a Checklist for Artificial Intelligence in Medical Imaging was proposed (99). In the batch of radiomics studies, few ones are able to provide clear details of the models and the selected predictors.

Moreover, reproducibility of radiomics features should be carefully explored. For instance, differences on imaging acquisition modalities could greatly influence radiomics features (100). Thus, harmonizing acquisition parameters between studies is a crucial step for future texture analysis (101). There is a real need for the harmonization of features to allow consistent findings in radiomics multicenter studies. Two main approaches could be considered to address this issue: harmonizing images and harmonizing radiomic features. The first one focuses on the harmonization issue in imaging and usually looks upon standardization of acquisition protocols and reconstruction settings, such as guidelines already available for PET/CT imaging (102). This approach should not be enough. Recently, techniques based on generative adversarial networks (103) have also been developed. Heterogeneous images are translated to match the statistical properties of a standard dataset, such as a template reference image. The second approach focuses on the issue in the feature area by either using prior feature selection based on their robustness, keeping only features insensitive to multicenter variability, or by keeping all features and harmonizing their statistical properties so they can be pooled during the modeling step. In this regard, different methods could be considered, such as normalization or batch-effect correction using the ComBat method.

One of the other challenges of imaging research is enhancing global collaboration and sharing trial data (104). Big and standardized clinical data will make radiomics clinically applicable (105). Access to big data is needed, as medical images are dispersed in different hospitals or data centers. Data sharing among institutes and hospitals is important for radiomics, although it presents complex logistical problems. The Cancer Imaging Archive (TCIA) provides a good example of data sharing with a large portion of clinical information  (106).

To perform generalizable models, it will be mandatory to develop them by involving multiple centers and to improve national and international collaboration (107).

Patient medical records are a great source of data. Some studies have shown the added value of clinical features combined with clinical ones. We can also hypothesize that the adjunction of genomic data and radiomics features from different imaging modalities could permit to get closer to a more personalized medicine.

The field is certainly high on promise and relatively low on data and proof, with the need of prospective validation (108). For clinical application, higher evidence levels are important. Prospective, multicenter, randomized controlled trials studies are needed.

One critical aspect of the radiomics workflow that remains relatively unexamined is the implementation of the software platforms used to calculate radiomics features. Some studies have demonstrated features variability from different software platforms (109, 110). The Image Biomarker Standardisation Initiative (IBSI), an international collaboration, was developed to help standardize radiomics feature calculation and has provided a framework to deliver practical solutions (111). The IBSI has made recommendations concerning feature calculation, standardized feature definition and nomenclature. A study (112) demonstrated the benefits of standardizing feature calculation platforms according to the IBSI with greater statistical reliability, but only when calculation settings were also harmonized.

Another point which should carefully assessed is the integration of radiomics software in the job process. Optimization, effectiveness and utility should be evaluated. As digital assistants (113), software programs designed to interact with people in a conversational manner, radiomics based software impact on clinical routine need assessment. By the same way, human factors (114) should be more consider as human factor interventions are known to have great potential to contribute to efficient Healthcare Information Technology design. Human factors and human-centered design play a critical role in ensuring that health IT is well designed and fits with clinical and patient workflows. The gaps between stakeholders, particularly vendors, researchers, clinicians, healthcare organization administrations, and purchasers, need also to be reduced.

## Conclusion

Radiomics in this last decade shows good ability to be considered as a potential new biomarker at different steps of the patient’s care in lung cancer. More multicentric prospective studies are still needed to evaluate the application of radiomics in daily practice. Deep learned radiomics should replace the traditional handcrafted radiomics for more efficiency on large datasets and more reproducibility.

## Author Contributions

RE wrote the manuscript. JB helped for article selection and manuscript redaction. JB, AB, NG, and PaG helped for technical review of the manuscript for machine learning and radiomics aspects. CD and PhG performed a review of the manuscript on the clinical aspects. All authors contributed to the article and approved the submitted version.

## Conflict of Interest

The authors declare that the research was conducted in the absence of any commercial or financial relationships that could be construed as a potential conflict of interest.

## Abbreviations

18F-FDG, 18F-fluorodeoxyglucose; ALK, anaplastic lymphoma kinase; ANN, artificial neural network; AUC, area under the curve; BRAF, v-raf murine sarcoma viral oncogene homolog B1; CAD, computer aided diagnosis; CNN, convolutional neural networks; CBCT, cone beam computed tomography; CT, computed tomography; EGFR, epidermal growth factor receptor; FNR, false negative rate; FPR, false positive rate; IBSI, Image Biomarker Standardisation Initiative; ICC, intraclass correlation coefficient; KRAS, Kirsten rat sarcoma viral oncogene homolog; LASSO, Least Absolute Shrinkage and Selection Operator; ML, machine learning; MRMR, minimum redundancy maximum relevance; NILST, National Lung Screening Trial; NSCLC, non-small-cell lung carcinoma; OS, overall survival; PCA, principal component analysis; pCR, pathologic complete response; PET, positron emission tomography; PML, pixel/voxel-based machine learning; PSO, particle swarm optimization; QDA, quadratic discriminant analysis; RFC, random forest classifier; ROI: region of interest; RP, radiation pneumonitis; RILI, radiation induced lung injury; RSF, random survival forests; SBRT, stereotactic body radiation therapy; SVM, support vector machine.
